# Sex-related differences in patients with coronavirus disease 2019: results of the Cardio-COVID-Italy multicentre study

**DOI:** 10.2459/JCM.0000000000001261

**Published:** 2022-01-31

**Authors:** Carlo Mario Lombardi, Claudia Specchia, Fabio Conforti, Maria Teresa La Rovere, Valentina Carubelli, Piergiuseppe Agostoni, Stefano Carugo, Gian Battista Danzi, Marco Guazzi, Andrea Mortara, Massimo Piepoli, Italo Porto, Gianfranco Sinagra, Maurizio Volterrani, Pietro Ameri, Massimiliano Gnecchi, Sergio Leonardi, Marco Merlo, Annamaria Iorio, Antonio Bellasi, Claudia Canale, Rita Camporotondo, Francesco Catagnano, Laura Adelaide Dalla Vecchia, Mattia Di Pasquale, Stefano Giovinazzo, Gloria Maccagni, Massimo Mapelli, Davide Margonato, Luca Monzo, Vincenzo Nuzzi, Chiara Oriecuia, Laura Pala, Giulia Peveri, Andrea Pozzi, Giovanni Provenzale, Filippo Sarullo, Marianna Adamo, Daniela Tomasoni, Riccardo Maria Inciardi, Michele Senni, Marco Metra

**Affiliations:** a Department of Cardiology, ASST Spedali Civili di Brescia and Department of Medical and Surgical Specialties, Radiological Sciences and Public Health, University of Brescia; b Department of Molecular and Translational Medicine, University of Brescia, Brescia; c Division of Melanoma, Sarcoma and Rare Tumors, European Institute of Oncology, Milan; d Istituti Clinici Scientifici Maugeri, IRCCS, Dipartimento di Cardiologia, Istituto Scientifico Montescano, Pavia; e Centro Cardiologico Monzino, IRCCS, Milan; f Department of Clinical Sciences and Community Health; g Division of Cardiology, Ospedale San Paolo, ASST Santi Paolo e Carlo, University of Milan, Milan; h Division of Cardiology, Ospedale di Cremona, Cremona; i Heart Failure Unit, Cardiology Department, University of Milan; j IRCCS San Donato Hospital, Milan; k Cardiology Department, Policlinico di Monza, Monza; l Heart Failure Unit, Guglielmo da Saliceto Hospital, AUSL Piacenza, Piacenza; m Institute of Life Sciences, Sant’Anna School of Advanced Studies, Pisa; n IRCCS Ospedale Policlinico San Martino – IRCCS Italian Cardiovascular Network, Department of Internal Medicine, University of Genova, Genova; o Cardiovascular Department, Azienda Sanitaria Universitaria Giuliano Isontina (ASUGI), and Department of Medical Surgical and Health Sciences, University of Trieste, Trieste; p Department of Cardiovascular and Respiratory Sciences, IRCCS, San Raffaele Pisana Rome, Rome; q Intensive Cardiac Care Unit, Fondazione IRCCS Policlinico S. Matteo; r Department of Molecular Medicine, Cardiology Unit, University of Pavia, Pavia; s Cardiology Unit, Cardiovascular Department, Papa Giovanni XXIII Hospital-Bergamo; t Innovation and Brand Reputation Unit, Papa Giovanni XXIII Hospital, Bergamo; u Istituti Clinici Scientifici Maugeri, IRCCS, Dipartimento di Cardiologia, Istituto Scientifico di Milano, Milan; v Department of Cardiology, University of Pavia, Pavia; w Istituto Clinico Casal Palocco; x Policlinico Casilino, Rome; y Department of Medical and Surgical Specialties, Radiological Sciences, and Public Health, University of Brescia, Brescia; z Cardiovascular Rehabilitation Unit, Buccheri La Ferla Fatebenefratelli Hospital, Palermo; aa Department of Medical and Surgical Specialties, Radiological Sciences and Public Health, University of Brescia and Cardiac Catheterization Laboratory and Cardiology, Cardio-thoracic Department ASST Spedali Civili, Brescia, Italy

**Keywords:** coronavirus study, inflammation, outcome, sex differences

## Abstract

**Introduction:**

The role of sex compared to comorbidities and other prognostic variables in patients with coronavirus disease (COVID-19) is unclear.

**Methods:**

This is a retrospective observational study on patients with COVID-19 infection, referred to 13 cardiology units. The primary objective was to assess the difference in risk of death between the sexes. The secondary objective was to explore sex-based heterogeneity in the association between demographic, clinical and laboratory variables, and patients’ risk of death.

**Results:**

Seven hundred and one patients were included: 214 (30.5%) women and 487 (69.5%) men. During a median follow-up of 15 days, deaths occurred in 39 (18.2%) women and 126 (25.9%) men. In a multivariable Cox regression model, men had a nonsignificantly higher risk of death vs. women (*P* = 0.07).

The risk of death was more than double in men with a low lymphocytes count as compared with men with a high lymphocytes count [overall survival hazard ratio (OS-HR) 2.56, 95% confidence interval (CI) 1.72–3.81]. In contrast, lymphocytes count was not related to death in women (*P* = 0.03).

Platelets count was associated with better outcome in men (OS-HR for increase of 50 × 10^3^ units: 0.88 95% CI 0.78–1.00) but not in women. The strength of association between higher PaO_2_/FiO_2_ ratio and lower risk of death was larger in women (OS-HR for increase of 50 mmHg/%: 0.72, 95% CI 0.59–0.89) vs. men (OS-HR: 0.88, 95% CI 0.80–0.98; *P* = 0.05).

**Conclusions:**

Patients’ sex is a relevant variable that should be taken into account when evaluating risk of death from COVID-19. There is a sex-based heterogeneity in the association between baseline variables and patients’ risk of death.

## Introduction

Sex and sex-based differences in prevalence and/or severity of a number of infectious diseases are largely known.^1^

On average, women have stronger innate and adaptive immune responses than males, and this results in faster clearance of pathogens and greater vaccine efficacy.^2^

Growing evidence suggests that sex-related differences also affect coronavirus disease (COVID-19).^3–6^

According to data available, it seems that women and men had similar susceptibility to get infected by COVID-19; however, there are relevant differences in the course of infection, risk of developing complications and mortality with an almost two-fold risk of death in males compared with women.^3,7^

However, the role of sex in Caucasian patients with COVID-19 is still unclear as no analysis has been done regarding its independent role compared with cardiac and noncardiac comorbidities.^3^

To our knowledge, only one study has investigated sex differences in the association with severity and mortality of COVID-19 in Chinese people.^8^

In our study, we assessed the independent role of sex compared to comorbidities and other prognostic variables in patients with COVID-19. This is the first study on Caucasian subjects investigating the role of sex as a determinant of outcomes and its interaction with different prognostic variables in a large cohort of patients with COVID-19.

## Methods

### Study design and participants

This a multicenter observational study on a retrospective cohort of consecutive adult patients with laboratory-confirmed COVID-19 infection, referred to 13 Italian Cardiology Units from 1 March to 9 April 2020. A confirmed case of COVID-19 was defined by a positive result on reverse-transcriptase-polymerase chain reaction (RT-PCR) assay of a nasopharyngeal swab. Patients hospitalized for cardiovascular reasons without a confirmed COVID-19 diagnosis were excluded.

Patients were followed up after the hospital admission and all-cause in-hospital mortality or discharge was ascertained until 23 April 2020.

The primary objective of this study was to assess the difference in risk of death between women and men. The secondary objective was to explore sex-based heterogeneity in the association between demographic, clinical or laboratory prognostic variables and patients’ risk of death.

This study complied with the edicts of the Declaration of Helsinki and was approved by the ethical committee of Spedali Civili di Brescia, Brescia, Italy (no. NP 4105).

### Data collection

Epidemiological, clinical and laboratory data of all patients were obtained from the electronic medical records of each designated hospital.

Detailed demographics information, comorbidities, symptoms, and disease severity of all patients were recorded or diagnosed on hospital admission. Laboratory examinations including routine blood tests; lymphocyte subsets; inflammatory or infection-related biomarkers; and cardiac, renal, liver, and coagulation function tests were obtained at initial diagnosis. Data regarding clinical treatment included COVID-19 specific therapy (oxygen therapy, nonmechanical and mechanical ventilation, antiviral agents, hydroxychloroquine, Tolicizumab, corticosteroids, antibiotic, anticoagulants) and background treatment. Coexisting comorbidities, chronic concomitant medications, as well as complications onset during the infection course were ascertained from medical records.

There were no cases lost to follow-up in this study.

### Statistical analyses

Data were presented stratified by sex. Continuous variables were shown as means and standard deviations, skewed variables as medians and interquartile ranges (IQR), and dichotomous variables as counts and percentages. Comparisons between two independent groups were made, respectively, using Student's *t*-test for means, Wilcoxon test for medians, and chi-squared test for proportions. For all variables with at least one expected count of less than 5, a Fisher's exact test instead of a chi-squared test was used.

Cumulative incidence function (CIF) of death was computed taking into account hospital discharge as a competing event. Comparison of CIFs among subgroups was performed by means of the Gray test. Variables clinically relevant or significantly associated with the risk of death at the univariable analysis were tested in a multiple Cox regression model to identify independent risk factors. The hazard ratios (HRs), 95% confidence intervals (CIs) and *P*-values from a Wald test were reported. Heterogeneity between HRs calculated for males and females was evaluated including in a Cox regression model of the interaction term between sex and the risk factor of interest. Models were adjusted for age, smoking and comorbidities.

A two-tailed *P*-value of <0.05 was considered statistically significant. Statistical analyses were performed using SAS statistical software version 9.4 (SAS Institute, Inc., Cary, NC, USA) and R version 3.6.1 (R Core Team 2019, Vienna, Austria).

## Results

Between 1 March and 9 April 2020, 701 patients with confirmed COVID -19 infection were admitted to the 13 hospitals included in our study; 214 (30.5%) were women and 487 (69.5%) men.

The demographic and clinical characteristics of the patients stratified by sex are shown in Table 1.

**Table 1:** Demographic and clinical characteristics of the study population at admission stratified by gender (*N* = 701)

	Female (*N* = 214)		Male (*N* = 487)		
	*N*		*N*		*P*-value
Age (years)	214	68.4 ± 14.0	487	66.7 ± 12.8	0.121
Body mass index ≥30 (kg/m^2^)	162	31 (19.1)	378	81 (21.4)	0.627
Smoker (ever)	182	39 (21.4)	410	123 (30.0)	0.040
Hypertension	211	120 (56.9)	485	278 (57.3)	0.979
Dyslipidaemia	211	53 (25.1)	484	140 (28.9)	0.348
Diabetes	211	42 (19.9)	485	120 (24.7)	0.197
Heart failure	211	24 (11.4)	485	69 (14.2)	0.371
Atrial fibrillation	211	35 (16.6)	485	71 (14.6)	0.587
Coronary artery disease	211	39 (18.5)	485	109 (22.5)	0.279
Prior cardiac surgery or percutaneous valve treatment	211	20 (9.5)	485	51 (10.5)	0.780
Prior heart transplantation/LVAD	211	0 (0.0)	485	4 (0.8)	0.320
Chronic obstructive pulmonary disease	211	23 (10.9)	485	45 (9.3)	0.601
Chronic kidney disease (eGFR <60 ml/min/m^2^)	211	45 (21.3)	485	83 (17.1)	0.225
Prior ACEi/ARB therapy	199	70 (35.2)	459	184 (40.1)	0.271
Prior BB therapy	198	70 (35.4)	458	180 (39.3)	0.385
Prior anticoagulant therapy	198	28 (14.1)	452	64 (14.2)	1.000
Prior statin therapy	198	54 (27.3)	460	127 (27.6)	1.000
Prior calcium antagonist therapy	200	52 (26.0)	460	116 (25.2)	0.909
Temperature (°C)	210	37.2 ± 0.9	478	37.3 ± 1.0	0.409
Fever (≥37.5°C)	210	85 (40.5)	478	211 (44.1)	0.417
Respiratory rate ≥22 (bpm)	182	0.0 (0.0–1.0)	357	1.0 (0.0–1.0)	0.151
SBP (mmHg)	212	129 ± 22	476	130 ± 22	0.383
DBP (mmHg)	212	73 ± 13	476	75 ± 13	0.065
Heart rate (bpm)	210	87 ± 20	477	87 ± 17	0.869
Oxygen saturation (ambient air, %)	209	94 (88–96)	478	92 (87–96)	0.071
PaO_2_/FiO_2_ (mmHg/%)	184	252 (153–326)	424	232 (119–314)	0.044
PaO_2_/FiO_2_ <300 (mmHg/%)	184	124 (67.4)	424	302 (71.2)	0.394
SOFA score	153	2 (1–3)	305	2 (2–3)	0.086
COVID score peak	39	5.0 (1.0–10.0)	132	9.0 (3.0–14.0)	0.012
LV ejection fraction (%)	82	56 (53–60)	183	55 (45–60)	0.010

Data shown as mean ± standard deviation, median (IQR) or count (%). ACEi, angiotensin-converting enzyme inhibitor; ARB, angiotensin receptor blocker; BB, beta blocker; COVID, coronavirus disease; DBP, diastolic blood pressure; eGFR, estimated glomerular filtration rate; FiO_2_, fraction of inspired oxygen; LV, left ventricular; LVAD, left ventricular assist device; PaO_2_, oxygen partial pressure at arterial gas analysis; SBP, systolic blood pressure; SOFA, sequential organ failure assessment.

When comparing women and men, we found no significant differences in patients’ age, body mass index and prevalence of comorbidities. The prevalence of smokers was slightly higher in men.

No differences were also found with respect to clinical characteristics at the time of hospitalization except for a lower PaO_2_/FiO_2_ ratio in men (median value: 232 mmHg/%, IQR: 119–314) as compared with women (median value: 252 mmHg/%, IQR: 153–326; *P* = 0.04).

Significant sex-based differences were observed in a number of laboratory analyses performed at the time of hospitalization (Table 2).

**Table 2:** Laboratory findings of the study population at admission stratified by gender (*N* = 701)

		Female (*N* = 214)		Male (*N* = 487)		
	Reference range	*N*		*N*		*P*-value
Red blood cell count (×10^6^/μl)	4.0–5.2	212	4.28 (3.77–4.69)	482	4.55 (4.10–5.93)	<0.001
Haemoglobin (g/dl)	12.0–16.0	211	12.4 (10.8–13.7)	480	13.6 (12.2–14.7)	<0.001
Haematocrit (%)	37.0–47.0	211	37.5 (32.9–40.3)	479	39.8 (36.1–43.2)	<0.001
White blood cell count (per μl)	4000–10 800	212	6610 (4715–9003)	482	6945 (5303–9598)	0.062
Lymphocytes absolute (per μl)	900–4000	189	1000 (710–1510)	436	900 (598–1183)	<0.001
Platelets count- (×10^3^/μL)	130–400	211	214 (168–294)	480	201 (151–262)	0.021
Serum creatinine (mg/dl)	0.60–1.00	207	0.80 (0.68–1.08)	478	1.02 (0.88–1.38)	<0.001
eGFR (CKD-EPI) ml/min	>80	207	74 (49–91)	478	76 (52–90)	0.931
Serum sodium (mEq/l)	136–145	209	138 (136–141)	475	138 (135–140)	0.069
Serum potassium (mEq/l)	3.4–4.5	209	3.9 (3.5–4.3)	472	4.0 (3.6–4.4)	0.047
Serum chloride (mEq/l)	98–107	154	101 (99–104)	356	100 (97–103)	0.071
CRP (mg/dl)	<5.0	198	36 (8–92)	455	67 (18–150)	<0.001
Procalcitonin (ng/ml)	<0.5	98	0.10 (0.05–0.26)	206	0.21 (0.09–0.85)	<0.001
Ferritin (μg/l)	30–400	105	396 (221–640)	231	915 (504–1729)	<0.001
D-dimer (ng/ml)	<232	144	871 (445–1864)	324	924 (468–2055)	0.746
Interleukin-6 (pg/ml)	<7.00	47	27 (11–54)	98	50 (19–98)	0.024
Troponin (elevated)		179	77 (43.0)	435	201 (46.2)	0.527
NT-proBNP (pg/ml)	<93	59	341 (98–875)	168	313 (99–1232)	0.680
Bilirubin (mg/dl)	<1.2	186	0.5 (0.3–0.6)	434	0.6 (0.5–0.8)	<0.001
Aspartate transaminase (U/l)	18–39	204	31 (21–47)	469	44 (30–69)	<0.001
Alanine transaminase (U/l)	10–50	206	23 (16–36)	470	37 (25–59)	<0.001
LDH (U/l)	135–225	163	305 (225–455)	403	377 (263–545)	<0.001
Creatine phosphokinase (U/l)	39–308	119	79 (41–158)	258	144 (65–355)	<0.001
Serum albumin (g/l)	45–52	110	3.2 (2.7–3.6)	272	3.2 (2.8–3.6)	0.980
INR	0.9–1.2	192	1.1 (1.0–1.2)	439	1.1 (1.0–1.2)	0.330
ABG test pH	7.37–7.45	190	7.46 (7.43–7.49)	428	7.47 (7.43–7.50)	0.339
ABG test lactate (mmol/l)	0.5–2.2	156	1.1 (0.8–1.4)	353	1.3 (0.9–1.7)	<0.001

Data shown as median (IQR) or count (%). ABG, arterial blood gas; CKD-EPI, chronic kidney disease epidemiology collaboration formula; CRP, C-reactive protein; eGFR, estimated glomerular filtration rate; INR, international normalized ratio; LDH, lactate dehydrogenase; NT-proBNP, N-terminal fragment of the prohormone brain natriuretic peptide.

As compared with men, women had higher levels of lymphocytes [median values (IQR) in women: 1000/μl (710–1510) vs. 900/μl (598–1183) in men; *P*-value <0.001], and platelets [214 000/μl (168 000–293 500) in women vs. 200 500/μl (151 000–262 250) in men; *P* = 0.02].

On the contrary, men had significantly higher levels of all the laboratory markers associated with systemic inflammation, including C-reactive protein (CRP, median value: 67 mg/dl vs. 36 mg/dl, *P* < 0.001), procalcitonin (median value: 0.21 ng/ml vs. 0.1 ng/ml; *P* < 0.001), Ferritin (median value: 915 μg/l vs. 396 μg/l; *P* < 0.001) and Interleukin-6 (median value: 50 pg/ml vs. 27 pg/ml; *P* = 0.02)

Table 3 reports details on in-hospital patients’ management and outcome according to sex. Regarding treatment, no differences were found between sexes, with the only exception being tocilizumab, which was administered more often in men than in women (13.5% vs. 7%; *P* = 0.02).

**Table 3:** In-hospital management and outcomes of the study population stratified by gender (*N* = 701)

	Female (*N* = 214)		Male (*N* = 487)		
	*N*		*N*		*P*-value
Hospital length of stay (days)	214	14.0 (9.0–23.0)	487	15.0 (9.0–25.0)	0.436
Pharmacological treatment					
Lopivanir/Ritonavir	213	51 (23.9)	483	138 (28.6)	0.241
Darunavir/Ritonavir	213	40 (18.8)	483	135 (28.0)	0.013
Remdesivir	213	1 (0.5)	483	4 (0.8)	1.000
Corticosteroid	213	101 (47.4)	483	244 (50.5)	0.502
Tocilizumab	213	15 (7.0)	483	65 (13.5)	0.021
Hydroxychloroquine	213	170 (79.8)	483	415 (85.9)	0.055
Antibiotics	213	179 (84.0)	483	432 (89.4)	0.060
Ventilatory support					
Oxygen support with FiO_2_ <50%	211	82 (38.9)	482	220 (45.6)	0.116
Oxygen support with FiO_2_ ≥50%	209	101 (48.3)	475	281 (59.2)	0.011
Noninvasive ventilation	211	76 (36.0)	484	226 (46.7)	0.011
Intubation	211	21 (10.0)	486	87 (17.9)	0.011
Complication					
ARDS	185	38 (20.5)	429	132 (30.8)	0.012
Sepsis	211	21 (10.0)	469	47 (10.0)	1.000
Acute renal insufficiency	151	14 (9.3)	342	58 (17.0)	0.037
Multiorgan failure	150	7 (4.7)	334	27 (8.1)	0.243
STEMI	211	2 (0.9)	477	9 (1.9)	0.518
NSTEMI	181	5 (2.8)	378	12 (3.2)	0.998
Heart failure	181	12 (6.6)	378	40 (10.6)	0.177
Ventricular arrhythmia	211	2 (0.9)	477	6 (1.3)	1.000
Pulmonary embolism	211	11 (5.2)	478	38 (7.9)	0.260
Other embolism	211	2 (0.9)	478	14 (2.9)	0.168
Stroke	211	2 (0.9)	478	1 (0.2)	0.224
Major bleeding	182	10 (5.5)	378	22 (5.8)	1.000
Delirium	149	6 (4.0)	334	12 (3.6)	1.000
Outcome					
Death	214	39 (18.2)	487	126 (25.9)	0.036
Cause of death^a^					
Respiratory insufficiency	39	28 (71.8)	123	98 (79.7)	0.418
Myocardial infarction	39	3 (7.7)	123	2 (1.6)	0.091
Pulmonary embolism	39	3 (7.7)	123	10 (8.1)	1.000
Stroke	39	0 (0.0)	123	4 (3.3)	0.573
Multiorgan failure	39	10 (25.6)	123	36 (29.3)	0.815
Bleeding	39	1 (2.6)	123	4 (3.3)	1.000

Data shown as median (IQR) or count (%).

a Multiple causes of death allowed. ARDS, acute respiratory distress syndrome; FiO_2_, fraction of inspired oxygen; NSTEMI, non-ST elevation myocardial infarction; STEMI, ST elevation myocardial infarction.

Despite similar demographic characteristics and treatments, major sex-based differences in outcomes were observed. As compared with women, a larger number of men required noninvasive ventilation (46.7% vs. 36%; *P* = 0.01) or intubation (17.9% vs. 10%; *P* = 0.01). Furthermore, a larger number of men developed major complications including ARDS (30.8% vs. 20.5%; *P* = 0.01) and acute kidney failure (17.3% and 9.3%, *P* = 0.04). Overall, 39 (18.2%) and 126 (25.9%) deaths occurred in women and in men respectively (*P* = 0.036).

Among 452 discharged patients, the median time from hospital admission to discharge was 14.5 days (IQR 9.0–23.0), whereas among 165 patients deceased during hospitalization, the median time to death was 10.0 days (IQR 6.0–17.0).

**Fig. 1:**
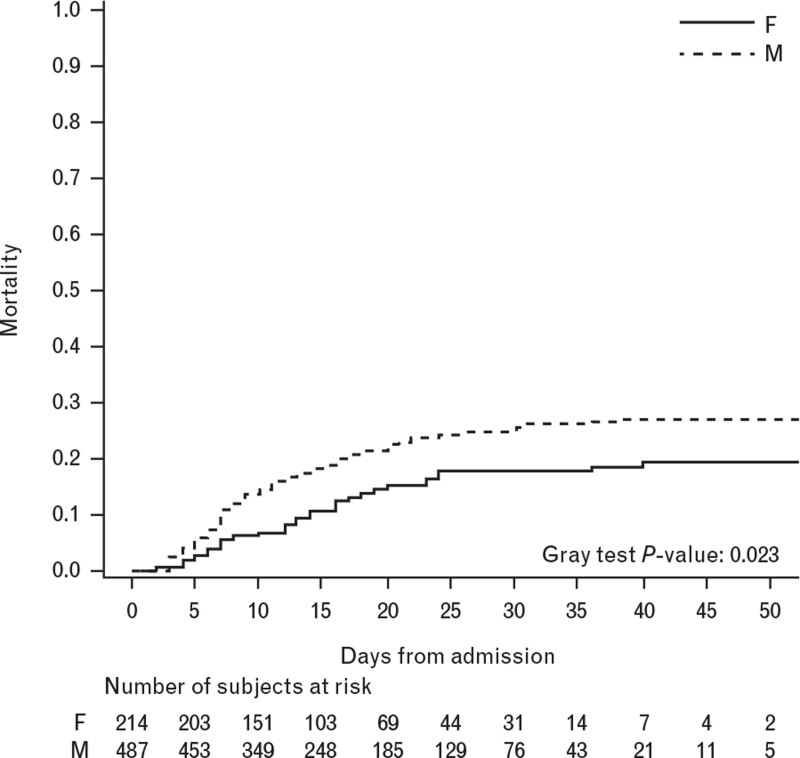
Cumulative incidence of death during hospitalization stratified by gender (*N* = 701).

During a median follow-up time of 15 days, the cumulative incidence of death, computed taking into account hospital discharge as a competing event, was higher among males than females during the follow-up (Gray test *P*-value: 0.023) (Fig. 1).

At univariate Cox regression analysis, a significantly higher risk of death was associated with demographic factors, including elderly age and smoking; laboratory variables, including lower levels of lymphocytes, red blood cells and oxygen saturation and higher levels of CRP, troponin and lactate dehydrogenase; and presence of several comorbidities, including hypertension, cardiovascular (CV) diseases, chronic obstructive pulmonary disease and chronic kidney disease. Using the multivariable Cox regression model, including all these variables, male patients had a nonsignificantly higher risk of death as compared with women [overall survival hazard ratio (OS-HR) 1.59, 95% CI 0.96–2.64 *P* = 0.07, Table 4].

**Table 4:** Univariable and multivariable Cox regression model for death

			Univariable		Multivariable (*N* = 459)	
	Level/units	*N*	HR (95% CI)	*P*-value	HR (95% CI)	*P*-value
Baseline variables						
Age	+5 years	701	1.36 (1.27–1.46)	<0.001	1.27 (1.13–1.42)	<0.001
Sex	M vs. F	701	1.35 (0.94–1.94)	0.099	1.59 (0.96–2.64)	0.074
Body mass index	≥30 vs. <30 (kg/m^2^)	540	1.22 (0.79–1.87)	0.376		
Smoker (ever)	Yes vs. No	592	1.45 (1.02–2.07)	0.040		
Respiratory rate	≥22 vs. <22	539	1.68 (1.16–2.43)	0.006		
SBP	+10 mmHg	688	0.94 (0.87–1.01)	0.084		
Oxygen saturation	+5%	687	0.83 (0.77–0.89)	<0.001	0.84 (0.75–0.95)	0.004
PaO_2_/FiO_2_	+50 mmHg/%	608	0.87 (0.81–0.94)	<0.001	0.90 (0.80–1.01)	0.060
SOFA	+1 point	458	1.39 (1.29–1.50)	<0.001		
Red blood cell count	+0.5 ×10^6^/μl	694	0.84 (0.75–0.94)	0.002		
White blood cell count	+1000 U/μl	694	1.03 (1.00–1.06)	0.026		
Lymphocytes	<900 vs. ≥900	625	2.12 (1.51–2.97)	<0.001	1.60 (1.04–2.47)	0.032
Platelets	+50 ×10^3^/μl	691	0.91 (0.83–0.99)	0.030	0.86 (0.76–0.98)	0.021
Creatinine	+1 mg/dl	685	1.13 (1.06–1.21)	<0.001		
eGFR (CKD-EPI)	+10 ml/min	685	0.82 (0.78–0.86)	<0.001	0.93 (0.84–1.03)	0.136
CRP	+10 mg/l	653	1.02 (1.01–1.04)	0.001	1.03 (1.01–1.05)	0.016
Procalcitonin	+0.5 ng/ml	304	1.00 (0.99–1.01)	0.666		
Ferritin	+100 μg/l	336	1.01 (1.00–1.02)	0.123		
D-dimer	+1000 ng/ml	468	1.02 (0.99–1.05)	0.167		
Interleukin-6	+10 pg/ml	145	1.00 (1.00–1.01)	0.215		
Troponin	Elevated vs. normal	614	3.22 (2.26–4.59)	<0.001	1.63 (1.06–2.50)	0.026
NT-proBNP	+1000 ng/l	227	1.03 (1.00–1.05)	0.036		
Bilirubin	+0.3 mg/dl	620	1.07 (0.97–1.19)	0.167		
LDH	+1000 mg/dl	566	1.12 (1.05–1.19)	<0.001		
Bilirubin	+0.3 mg/dl	620	1.07 (0.97–1.19)	0.167		
INR	+1	631	1.22 (1.03–1.44)	0.024		
ABG test lactate	+1 mmol/l	509	1.22 (1.14–1.30)	<0.001		
Comorbidities						
Hypertension	Yes vs. no	696	1.91 (1.37–2.67)	<0.001	1.11 (0.70–1.75)	0.657
Diabetes	Yes vs. no	696	1.34 (0.95–1.87)	0.096		
Heart failure	Yes vs. no	696	2.45 (1.70–3.52)	<0.001	1.97 (1.16–3.36)	0.013
Atrial fibrillation	Yes vs. no	696	2.48 (1.74–3.53)	<0.001	1.27 (0.76–2.12)	0.361
Coronary artery disease	Yes vs. no	696	2.28 (1.65–3.16)	<0.001	1.09 (0.69–1.72)	0.722
Chronic obstructive pulmonary disease	Yes vs. no	696	1.76 (1.14–2.71)	0.011	1.50 (0.86–2.63)	0.154
Chronic kidney disease	Yes vs. no	696	2.80 (2.03–3.87)	<0.001	0.89 (0.51–1.56)	0.687
Medication history						
Prior ACEi-ARBS therapy	Yes vs. no	658	1.56 (1.14–2.13)	0.006		
Prior BB therapy	Yes vs. no	656	1.99 (1.45–2.72)	<0.001		
Prior statin therapy	Yes vs. no	658	1.88 (1.36–2.61)	<0.001		
Prior calcium antagonists therapy	Yes vs. no	660	1.39 (0.99–1.95)	0.055		

ABG, arterial blood gas; ACEi, angiotensin-converting enzyme inhibitor; ARB, angiotensin receptor blocker; BB, beta blocker; CKD-EPI, chronic kidney disease epidemiology collaboration formula; CRP, C-reactive protein; eGFR, estimated glomerular filtration rate; FiO_2_, fraction of inspired oxygen; INR, international normalized ratio; LDH, lactate dehydrogenase; NT-proBNP, N-terminal fragment of the prohormone brain natriuretic peptide; PaO_2_, oxygen partial pressure at arterial gas analysis; SBP, systolic blood pressure; SOFA, sequential organ failure assessment.

**Fig. 2:**
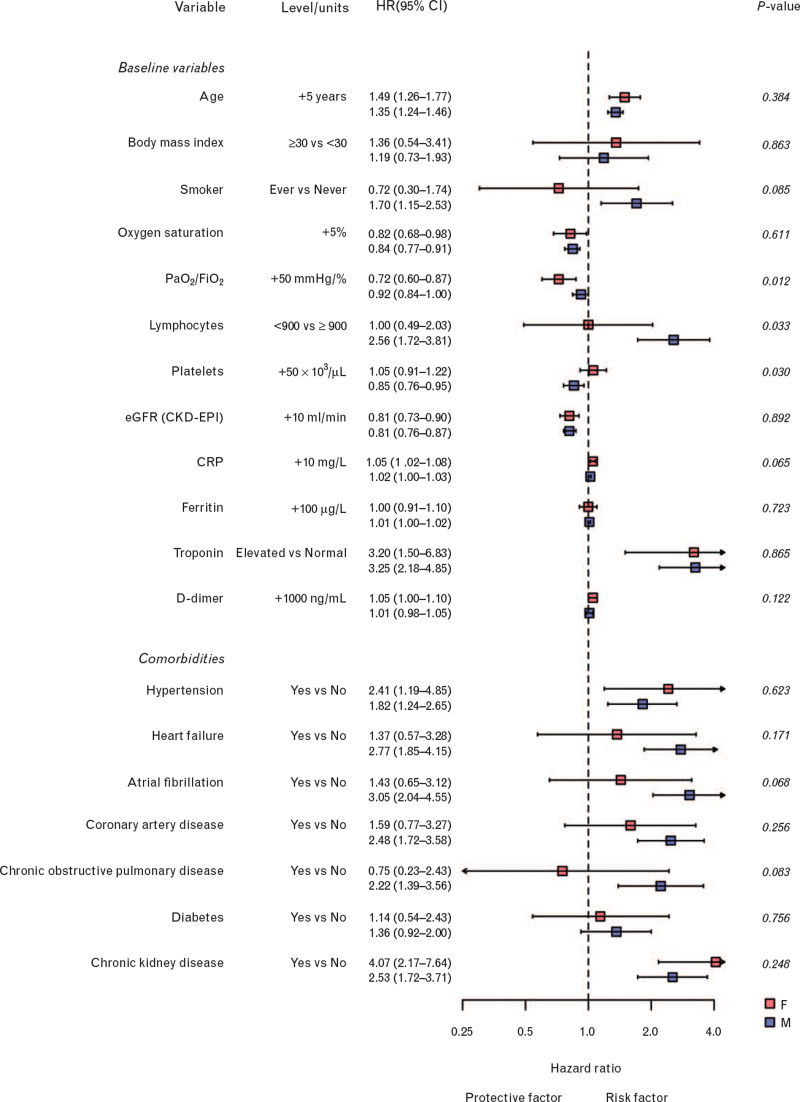
Forest plot comparing association of baseline variables and comorbidities with risk of death between females and males.

A significant interaction with sex was found in the association between patients’ risk of death and some laboratory variables, namely lymphocytes count, platelets count and PaO_2_/FiO_2_ ratio (Fig. 2).

**Fig. 3:**
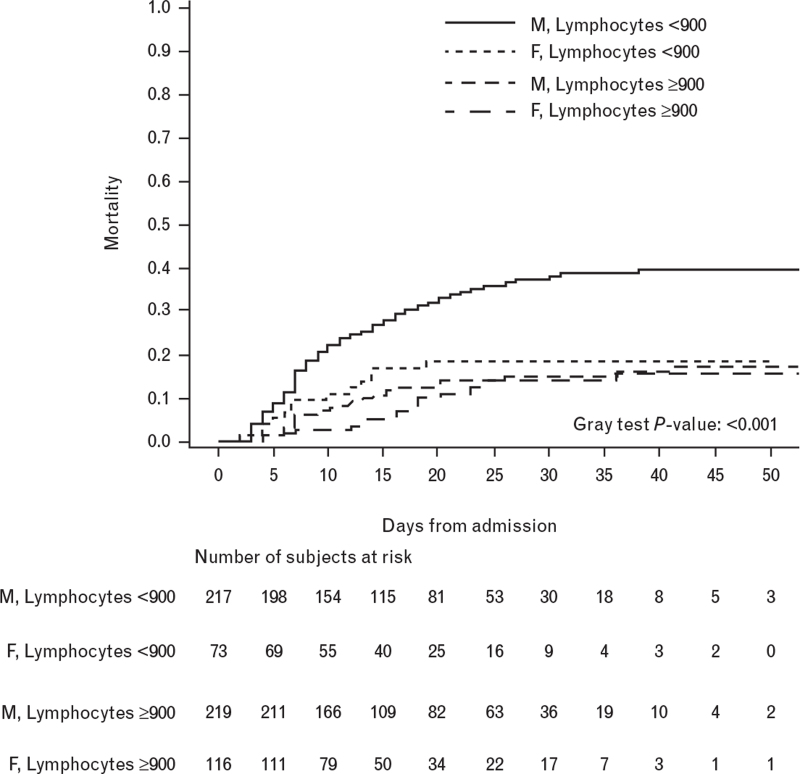
Cumulative incidence of death during hospitalization stratified by gender and lymphocytes at admission (*N* = 625).

The risk of death was more than double in male patients with a low lymphocytes count (i.e. below the median value of 900/μl) as compared with men with a high lymphocytes count (i.e. above 900/μl; OS-HR 2.56, 95% CI 1.72–3.81), whereas it was not different in women with low or high lymphocytes counts (OS-HR 1.00, 95% CI 0.49–2.03; *P* for heterogeneity = 0.03).

Such interaction remained significant on multivariable Cox regression analysis after adjusting, respectively, for patients’ age (*P* for heterogeneity = 0.04) or age and comorbidities (*P* for heterogeneity = 0.03) or age, comorbidities and patients’ smoking habitus (*P* for heterogeneity = 0.05) (Table 5).

**Table 5:** Unadjusted and adjusted Cox regression HRs (95% CIs) for death stratified by sex, and *P*-values for heterogeneity between strata

		Unadjusted		Adjusted for age		Adjusted for age and comorbidities^a^		Adjusted for age, smoke and comorbidities^a^	
	Sex	HR (95%CI)	*P*-value	HR (95% CI)	*P*-value	HR (95%CI)	*P*-value	HR (95% CI)	*P*-value
Platelets	F	1.05 (0.91–1.22)	0.030	1.05 (0.91–1.22)	0.029	1.05 (0.91–1.22)	0.031	1.07 (0.92–1.25)	0.091
	M	0.85 (0.76–0.95)		0.86 (0.77–0.96)		0.86 (0.77–0.96)		0.88 (0.78–1.00)	
Lymphocytes (<900 vs. ≥900)	F	1.00 (0.49–2.03)	0.033	1.00 (0.49–2.05)	0.039	1.01 (0.49–2.06)	0.033	1.18 (0.56–2.49)	0.051
	M	2.56 (1.72–3.81)		2.44 (1.64–3.63)		2.49 (1.66–3.73)		2.60 (1.65–4.10)	
PaO_2_/FiO_2_	F	0.72 (0.60–0.87)	0.012	0.69 (0.56–0.85)	0.024	0.69 (0.56–0.85)	0.022	0.72 (0.59–0.89)	0.053
	M	0.92 (0.84–1.00)		0.88 (0.80–0.96)		0.88 (0.80–0.97)		0.88 (0.80–0.98)	

CI, confidence interval; HR, hazard ratio.

a At least one of: obesity, hypertension, diabetes, heart failure, atrial fibrillation, coronary artery disease, chronic obstructive pulmonary disease, or chronic kidney disease.

Similarly, a higher platelets count was associated with a reduction in the risk of death in men (OS-HR for increase of 50 × 10^3^ units: 0.88, 95% CI 0.78–1.00) but not in women (1.07, 95% CI 0.92–1.25) although this interaction did not reach statistical significance (adjusted *P* for heterogeneity = 0.09). The association between a higher PaO_2_/FiO_2_ ratio and better survival was larger in women (OS-HR for increase of 50 mmHg/%: 0.72, 95% CI 0.59–0.89) as compared with men (OS-HR 0.88, 95% CI 0.80–0.98; adjusted *P* for heterogeneity = 0.05).

There were also trends for a different impact of cardiovascular comorbidities on patients’ outcome according to sex, although no significant difference was found (Fig. 2).

## Discussion

In this large multicenter retrospective cohort analysis, the risk of death from COVID-19 was numerically higher in men as compared with women. Men also had an increased rate of untoward events or complications, including invasive ventilation, ARDS and acute renal failure. These differences in outcomes were found despite similar demographic and clinical characteristics, including comorbidities and treatment between the two sexes.

Different mortality rates between men and women affected by COVID-19 have been reported.^3–7^ Several explanations have been postulated to account for such a difference, ranging from sex differences in immune responses to mere association with a different prevalence of chronic comorbidities or different behaviors in men and women.^3^

In this analysis, we have not found differences between men and women regarding the prevalence of major cardiovascular and respiratory comorbidities, and also the pharmacological management of COVID-19 was similar in the two groups.

This finding is in keeping with a recent national Italian registry showing no significant differences in these relevant clinical variables according to sex, but a worse prognosis for male patients.^7^

The statistically significant and clinically meaningful difference in mortality was also confirmed after adjusting for other factors not balanced between the two groups, and potentially affecting patients’ prognosis, such us smoking habitus.

In addition, we identified differences in a number of relevant factors associated with innate and adaptive immune responses between men and women, including different degrees of lymphocytopenia and blood levels of markers of systemic inflammation and showed a significant sex-based heterogeneity in their prognostic role.

Our results support the hypothesis of the importance of biological intrinsic factors, such as differences in immune responses, in the pathogenesis of sex-based dimorphism of the course and severity of COVID-19 infection.

New findings recently published in the *Nature* journal, further support such a hypothesis, revealing several molecular differences in immune responses during the disease course of severe acute respiratory syndrome coronavirus 2 (SARS-CoV-2) infection in male and female patients. It has been shown that several key elements of innate response are greater in men, such as higher plasma levels of IL-8 and IL-18 immune cytokines, whereas women mounted significantly more robust T cell activation during SARS-CoV-2 infection.^9^

Relevant differences of both innate and adaptive immune responses between men and women explain the different prevalence and mortality from infectious and autoimmune diseases and from several types of cancers.^1,2^

These sex-based differences of immune responses reflect complex interactions among genes, hormones, and the environment.^1,2^

The X chromosome contains a large number of immune-related genes.^10^

Immune-related genes encoded on the X chromosome may escape X inactivation, resulting in higher expression levels in women than men.^1,2^

Sex hormones modulate the development and function of multiple immune cell populations.^1,2^ Putative androgen response elements (AREs) and estrogen response elements (EREs) are present in the promoters of several innate and adaptive immune genes, suggesting that sex steroids may directly regulate their expression.^1,2^

In-vivo studies showed that male mice were more susceptible to COVID-19 infection. Hormonal suppression through an oestrogen receptor antagonist or ovariectomy increased mortality in female mice, demonstrating the protective role of oestrogens against COVID-19.^11^

The COVID-19 spike protein binds to the ACEII receptor on human cells, and the human protease TMPRSS2 activates the spike protein and allows the viral entry, being paramount for viral spread and pathogenesis in the infected host.^12^

The human TMPRSS2 gene promoter has an ARE and androgens are positive regulators of its transcription.^13^ An allelic variant predicted to induce higher levels of TMPRSS2 has been recently found as being more frequent in the Italian than in the East Asian population.^14^

Although its relation with tissue levels may vary, plasma levels of the COVID-19 receptor in human cells, ACE2, were significantly higher in males compared with females in a recent study of patients with heart failure, consistently with the increased susceptibility and more severe clinical course of COVID-19 in men.^15^ Taken together all these intrinsic biological factors could explain the higher virulence of COVID-19 in men.^13^

Obviously, a different prevalence of comorbidities could further contribute to the worsening of the prognosis for men. Indeed, in our cohort of patients, chronic CV comorbidities had a significant association with worse prognosis, particularly in male patients.

### Limitations

The retrospective nature of our analyses is the major limitation of the study.

However, the availability of individual data of a large cohort of patients allowed us to adjust analyses for sex-related differences in the most relevant variables associated with patients’ prognosis, including age, comorbidities and smoking status.

Furthermore, we did not have data on the menopausal status of women as well as on hormonal replacement therapy in postmenopausal women, preventing the possibility to assess the association between sex-hormonal status and the prognosis of female patients with COVID-19.^16^

## Conclusion

In conclusion, our results demonstrate that sex is a variable that may influence patients’ prognosis in COVID-19 independently from other known factors, particularly comorbidities and smoking habitus.

Furthermore, they also highlight the need to take patients’ sex into account when evaluating the risk of death from COVID-19, not only because it is a meaningful independent prognostic factor, but also because there is a relevant sex-based heterogeneity in the association between several other factors and patients’ risk of death. Future research should investigate the biological mechanisms that drive the pathogenesis of the sex-based dimorphism of COVID-19 virulence.

Indeed, to identify the molecular mechanisms underlying the different prognosis between men and women could have relevant implications, including the possibility to tailor specific preventive strategies and therapeutic approaches for women and men, in order to improve outcomes for both.

## Acknowledgements

Author contributions: Marco Metra, Carlo Lombardi and Claudia Specchia had full access to all of the data in the study and take responsibility for the integrity of the data and the accuracy of the data analysis. Carlo Lombardi and Claudia Specchia contributed equally to this work and are co-first authors.

Author contributions: V.C. received consulting honoraria from CVie Therapeutics Limited, Servier, and Windtree Therapeutics.

P.A. received speaker and advisor honoraria from Novartis, AstraZeneca, Vifor, Daiichi-Sankyo, Boehringer Ingelheim, Pfizer, GSK and MSD.

A.M. reports personal consulting honoraria from Novartis, Servier, Astra Zeneca for participation in advisory board meetings and receives grants from Novartis and Niccomo for research trials.

M.P. received a research grant and speaking fees from Novartis, Servier, Vifor.

M.M. reports personal consulting honoraria from Bayer, Novartis, Fresenius, Servier, and Windtree Therapeutics for participation to advisory board meetings and executive committees of clinical trials.

Funding: This research was not supported by any public or private funding.

### Conflicts of interest

There are no conflicts of interest.

Additional information: List of centers and collaborators: Additional Supporting Information may be found in Appendix S1.
